# Synthesis, Characterization, and Antimicrobial Activity of Novel Sulfonated Copper-Triazine Complexes

**DOI:** 10.1155/2018/2530851

**Published:** 2018-08-29

**Authors:** Supun Katugampala, Inoka C. Perera, Chandrika Nanayakkara, Theshini Perera

**Affiliations:** ^1^Department of Chemistry, University of Sri Jayewardenepura, Nugegoda, Sri Lanka; ^2^Department of Zoology and Environment Science, University of Colombo, Colombo, Sri Lanka; ^3^Department of Plant Science, University of Colombo, Colombo, Sri Lanka

## Abstract

Metallotriazine complexes possess interesting biological and medicinal properties, and the present study focuses on the synthesis, characterization, and antimicrobial activity of four novel copper-triazine derivatives in search of potent antibacterial and antifungal drug leads. In this study, 3-(2-pyridyl)-5,6-diphenyl-1,2,4-triazine-4,4′-disulfonic acid monosodium salt (L1, ferrozine) and 3-(2-pyridyl)-5,6-di(2-furyl)-1,2,4-triazine-5,5′-disulfonic acid disodium salt (L2, ferene) have been used as ligands to study the complexation towards copper(II). The synthesized complexes, [CuCl_2_(ferrozine)]·7H_2_O·MeOH (**1**), [CuCl_2_(ferrozine)_2_]·5H_2_O·MeOH (**2**), [CuCl_2_(ferene)]·H_2_O·MeOH (**3**), and [CuCl_2_(ferene)_2_]·H_2_O·MeOH (**4**), have been characterized spectroscopically, and preliminary bioassays have been carried out. FTIR spectroscopic data have shown that N=N and C=N stretching frequencies of complexes have been shifted towards lower frequencies in comparison with that of the ligands, confirming new bond formation between Cu and N, which in turn lowers the strength of N=N and C=N bonds. In addition, a bathochromic shift has been observed for UV-visible spectra of complexes (**1**), (**2**), (**3**), and (**4**). Furthermore, elemental analysis data have been useful to obtain empirical formulas of these complexes and to establish the purity of each complex. Complexes (**1**) and (**2**) have shown antibacterial activity for both *S. aureus* (ATCC® 25923) and *E. coli* (ATCC® 25922) at 1 mg/disc concentration, and ferrozine has shown a larger inhibition zone against the clinical sample of *C. albicans* at 1 mg/disc concentration in comparison with the positive control, fluconazole.

## 1. Introduction

Transition metals have numerous and unique biological, chemical, and physical properties due to the availability of d electrons in valance shells. Much attention has been focused on copper complexes due to their various potential biological activities [1–4] out of which antimicrobial [5] and antiviral activities is paramount [6–15].

Since triazine is a well-known natural material which possesses many biological properties [16–21], it is not surprising that organometallic complexes of triazine with first row transition metals (Mn [22, 23], Co [24, 25], Ni [24, 25], Cu [22, 24–28], and Zn [25]), with second row transition metals (Ru [29], Pd [30], Ag [31], and Cd [32]), and with third row transition metals (Re [33] and Pt [34–36]) have been synthesized, and their activities explored as catalysts [37] and biological agents such as antibacterial [25], anticancer [29, 36], antifouling [24], antifungal [33], anti-HIV [35], antimicrobial [25], antiproliferative [26, 34], antiviral [28, 35], and DNA binding [26, 29, 30] agents.

**Figure 1:**
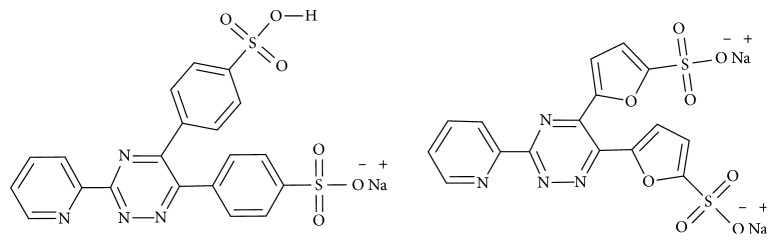
Structure of 3-(2-pyridyl)-5,6-diphenyl-1,2,4-triazine-*p*,*p*′-disulfonic acid monosodium salt (L1) (a) and 3-(2-pyridyl)-5,6-di(2-furyl)-1,2,4-triazine-5,5′-disulfonic acid disodium salt (L2) (b).

Even though many reports exist of metal complexes of triazine derivatives as detailed above, metal complexes containing the pyridyl-1,2,4-triazine core are relatively unexplored. Platinum(II) complexes of sulphonated 2-pyridyl-1,2,4-triazine have been reported to possess anti-HIV activity [35]. A copper(II) complex bearing 2,4,6-tris(2-pyridyl)-1,3,5-triazine ligand has been reported to bind DNA in a moderately strong way exhibiting significantly better anticancer activity against breast cancer in comparison with cisplatin [26]. An octahedral complex of rhenium(V), ML1L2L3L4 (where L1 = oxo, L2 = chloride, L3 = triphenylphosphine, and L4 = 3-hydrazino-5,6-diphenyl-1,2,4-triazine), has shown comparable antifungal activity against *Alternaria alternata* and *Aspergillus niger* [33]. We ourselves have explored the potential of using rhenium complexes of ferene and ferrozine (Figure 1) as biological imaging agents [38]. In our most recent work, we have commented on the possible use of the scaffold of sulfonated pyridyl triazine complexes being utilized as serum albumin transporters [39]. As such, it seems prudent to now explore its binding towards copper.

**Figure 2:**
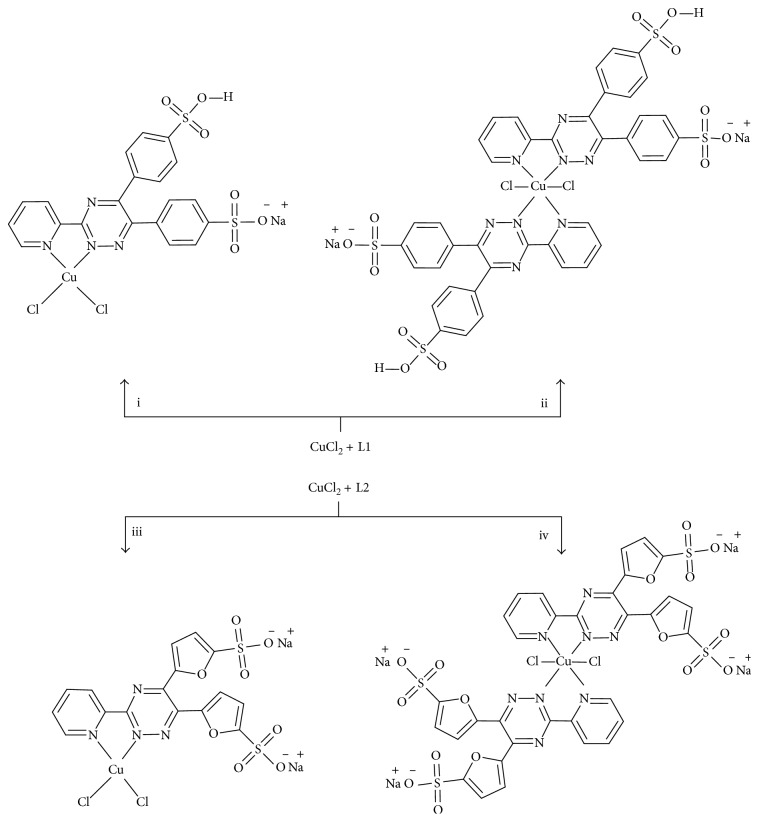
Synthetic routes for ML1Cl_2_ (complex (**1**)) (i), M(L1)_2_Cl_2_ (complex (**2**)) (ii), ML2Cl_2_ (complex (**3**)) (iii), and M(L2)_2_Cl_2_ (complex (**4**)) (iv) complexes. NB: L1 = 3-(2-pyridyl)-5,6-diphenyl-1,2,4-triazine-*p*,*p*′-disulfonic acid monosodium salt; L2 = 3-(2-pyridyl)-5,6-di(2-furyl)-1,2,4-triazine-5,5′-disulfonic acid disodium salt. Solvent molecules in complexes (**1**)–(**4**) have been omitted for clarity. Molar ratios of reactants: (i) CuCl_2_ : L1 = 1 : 1, (ii) CuCl_2_ : L1 = 1 : 2, (iii) CuCl_2_ : L2 = 1 : 1, and (iv) CuCl_2_ : L2 = 1 : 2.

Thus, the current study explores the synthesis of four novel water-soluble complexes of the type, ML_*n*_Cl_2_ (Figure 2) (where M = Cu^2+^, L = 3-(2-pyridyl)-5,6-diphenyl-1,2,4-triazine-4′,4″-disulfonic acid sodium salt/3-(2-pyridyl)-5,6-di(2-furyl)-1,2,4-triazine-5′,5″-disulfonic acid disodium salt, and *n *= 1/2), their chemical characterization, and preliminary tests to assess antimicrobial activity of above synthesized complexes as well as of the ligands.

## 2. Experimental

### 2.1. Materials Used

All chemicals and reagents used for the synthesis were commercially available and used without further purification. 3-(2-Pyridyl)-5,6-diphenyl-1,2,4-triazine-4,4′-disulfonic acid monosodium salt (ferrozine), 3-(2-pyridyl)-5,6-di(2-furyl)-1,2,4-triazine-5,5′-disulfonic acid disodium salt (ferene), and methanol ACS reagent (assay ≥99.8%) were purchased from Sigma-Aldrich, and copper(II) chloride dihydrate was purchased from Research-Lab Fine Chem Industries. Mueller-Hinton agar was purchased from Hardy Diagnostics, USA. Sodium chloride, sodium hydroxide, and dextrose were purchased from HiMedia Laboratories. The bacteria were obtained by the Industrial Technology Institute, Colombo.

### 2.2. Instrumentation

Elemental analysis was carried out on PerkinElmer 2400 Series II CHNS/O Elemental Analyzer at Atlantic Microlabs, USA. IR spectra were recorded using Thermo Scientific NICOLET iS10 spectrophotometer in the spectral range 4000–650 cm^−1^ for both ligands and complexes. Thermo Spectronic Helios alpha UV-Vis double-beam spectrophotometer was used to measure the absorbance in the range of 190–1100 nm, and baseline correction was performed using matched quartz cuvettes. High-resolution mass spectra were recorded on an Agilent 6210 ESI TOF LCMS mass spectrometer.

### 2.3. Synthesis

#### 2.3.1. Preparation of [CuCl_2_(ferrozine)]·7H_2_O·MeOH (**1**)

A solution of ferrozine (0.25 mmol, 0.1269 g) in methanol (8.0 cm^3^) was added to copper chloride dihydrate (0.25 mmol, 0.0435 g) in methanol (2.0 cm^3^). Then the resulting mixture was stirred for 2 hours at room temperature and progression of reaction checked using TLC. A light green colour crystalline precipitate was obtained after 2 days and collected by filtration (yield: 0.1264 g, 64%). IR (ATR; *ν*/cm^−1^): 1596.84(m) and 1498.22(s), *ν*_C=N_ and *ν*_N=N_. UV-Vis (MeOH; *λ*_max_ [nm]): 205, 242, 298, and 327. Anal. Calc. for C_20_H_13_Cl_2_CuN_4_NaO_6_S_2_·7H_2_O·CH_3_OH: C, 32.12; H, 3.98; N, 7.14. Found: C: 31.68%, H: 3.80%, and N: 7.42%. ESI-MS (*m*/*z*): [M − H]^−^ calcd for C_20_H_13_ClCuN_4_O_6_S_2_, 565.9179; found, 565.9188.

#### 2.3.2. Preparation of [CuCl_2_(ferrozine)2]·5H_2_O·MeOH (**2**)

A procedure similar to that given above was followed using copper chloride dihydrate (0.25 mmol, 0.0435 g) and ferrozine (0.50 mmol, 0.2538 g). The resulting mixture was stirred for 5 hours. A dark green colour crystalline precipitate was obtained after 2 days and collected by filtration (yield: 0.1937 g, 62%). IR (ATR; *ν*/cm^−1^): 1595.69(m) and 1498.50(s), *ν*_C=N_ and *ν*_N=N_. UV-Vis (MeOH; *λ*_max_ [nm]): 213, 240, 301, and 334. Anal. Calc. for C_40_H_26_Cl_2_CuN_8_Na_2_O_12_S_4_·5H_2_O·CH_3_OH: C, 39.66; H, 3.25; N, 9.03. Found: C: 39.29%, H: 3.76%, N: 9.23%. ESI-MS (*m*/*z*): [M − H]^−^ calcd for C_40_H_26_CuN_8_O_12_S_4_, 999.9833; found, 999.9776.

#### 2.3.3. Preparation of [CuCl_2_(ferene)]·H_2_O·MeOH (**3**)

A solution of ferene (0.25 mmol, 0.1236 g) in methanol (8.0 cm^3^) was added to copper chloride dihydrate (0.25 mmol, 0.0435 g) in methanol (2.0 cm^3^). Then the resulting mixture was stirred for 6 hours at room temperature and progression of reaction checked using TLC technique initially and at the end. A yellow colour crystalline precipitate was obtained after 1 day and collected by filtration (yield: 0.1183 g, 75%). IR (ATR; *ν*/cm^−1^): 1567.49(m) and 1499.15(s), *ν*_C=N_ and *ν*_N=N_. UV-Vis (MeOH; *λ*_max_ [nm]): 202, 239, 338, and 371. Anal. Calc. for C_16_H_8_Cl_2_CuN_4_O_8_S_2_·H_2_O·CH_3_OH: C, 32.16; H, 2.54; N, 8.82. Found: C: 32.12%, H: 2.76%, N: 9.29%. ESI-MS (*m*/*z*): [M]^−^ calcd for C_16_H_8_CuN_4_O_8_S_2_, 510.9085; found, 510.9084.

#### 2.3.4. Preparation of [CuCl_2_(ferene)_2_]·H_2_O·MeOH (**4**)

A procedure similar to above was followed using copper chloride dihydrate (0.25 mmol, 0.0435 g) and ferene (0.50 mmol, 0.2472 g). The resulting mixture was stirred for 5 hours. A brown-yellow colour crystalline precipitate was obtained after 1 day and collected by filtration (yield: 0.1912 g, 65%). IR (ATR; *ν*/cm^−1^): 1569.82(m) and 1494.40(s), *ν*_C=N_ and *ν*_N=N_. UV-Vis (MeOH; *λ*_max_ [nm]): 208, 246, 338 and 371. Anal. Calc. for C_32_H_16_Cl_2_CuN_8_Na_4_O_16_S_4_·H_2_O·CH_3_OH: C, 33.78; H, 1.89; N, 9.56. Found: C: 33.76%, H: 2.42%, N: 9.58%.

### 2.4. Antimicrobial Assay

Compounds were tested against Gram-positive *Staphylococcus aureus* ATCC® 25923 and Gram-negative *Escherichia coli* ATCC® 25922 bacterial species and a clinical isolate of *Candida albicans* as a fungal species. Antimicrobial assay was performed by a standard disk diffusion assay [40] where the inhibition zones were measured and expressed as a mean of three replicates. Gentamycin and flucanazole were used as positive controls, and methanol was used as the negative control.

## 3. Results and Discussion

### 3.1. Synthesis

Copper chloride and the relevant ligands were used in 1 : 1 and 1 : 2 ratios to synthesize the desired metal complexes (Figure 2). Thin-layer chromatography (TLC) was initially used to monitor the progress of reaction, and visualization of spots was done using an iodine bath.

### 3.2. FTIR Analysis

FTIR data were recorded for dried crystals of ligands and complexes (**1**)–(**4**), and literature values were utilized where relevant [41]. The stretching frequency of the pyridine ring (*ν*_C=N_) and stretching frequency of the triazine ring (*ν*_N=N_) are considered mostly, because their values change upon formation of new bonds serving as good indicators of complex formation.

Stretching frequencies of N=N and C=N in complexes (**1**) and (**2**) have shifted to lower frequencies as expected, compared to those values of the free ferrozine ligand, due to *σ* donation of N lone pair which lowers strength of N=N and C=N bonds (Table 1). Furthermore, a broad band around 3400–3300 cm^−1^ was observed due to OH groups from methanol or water.

Similarly, stretching frequencies of N=N and C=N in complexes (**3**) and (**4**) were observed at lower frequencies in comparison with those of the free ferrozine ligand (Table 1), and a broad band was observed around 3400–3300 cm^−1^ due to OH groups of solvent.

### 3.3. UV-Visible Spectroscopy

**Figure 3:**
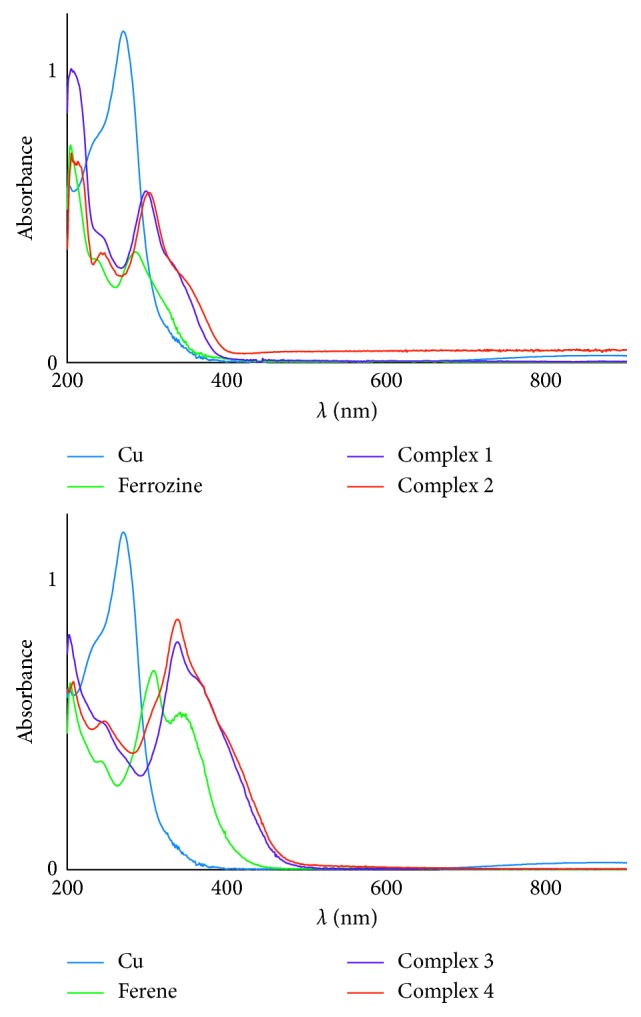
UV-visible spectra recorded in methanol of ferrozine, complexes (**1**) and (**2**) (a) and ferene, complexes (**3**) and (**4**) (b).

UV-Vis spectra of reactants and complexes (**1**, **2**, **3**, and **4**) were recorded in methanol at room temperature (Figure 3, Table S1, Supplementary Materials). The absorption wavelengths of complexes (**1**)–(**4**) have shifted towards longer wavelengths (bathochromic shift) compared to the wavelengths of the reactants (copper, ferrozine, and ferene). Both ferrozine and ferene have aromatic ring systems, and *π*–*π* ^*∗*^ transitions are thus possible [42]. These results are in agreement with those previously reported for zinc complexes of ferene and ferrozine [39] where a bathochromic shift was observed for both mono and bis complexes in comparison with that of the free ligand.

### 3.4. Elemental Analysis

Empirical formulas related to experimental values aided in obtaining the exact molecular formulas of all four complexes (Table 2). It can be seen that experimental values are within ±0.4% of expected values indicating purity of the synthesized complexes.

### 3.5. Antimicrobial Activity

All four complexes and ligands were studied *in vitro* for their antimicrobial activity against Gram-positive *Staphylococcus aureus* ATCC® 25923 and negative bacteria *Escherichia coli* ATCC® 25922 as well as the unicellular fungal species, *Candida albicans*. Inhibition zones were obtained by adding a concentration of 1 mg/disc, and the diameters of the zones are given in Table 3 for bacteria and Table 4 for fungi.

Analysis of the inhibition zone diameter revealed that only complex (**1**) and complex (**2**) show moderate antibacterial activity when compared to the positive control. It is interesting to see that ferrozine ligand demonstrates antifungal activity.

Antimicrobial activity reported here is of moderate value. Further studies are warranted to optimize this system for greater activity.

## 4. Conclusions

We have described the synthesis of four novel water-soluble copper complexes bearing sulfonated pyridyl triazine ligands. FTIR spectroscopic data have confirmed the existence of Cu-N bonds in all four complexes because stretching frequencies of N=N and C=N complexes have been shifted towards lower frequencies in comparison with that of the ligands. In UV-Vis spectra, a bathochromic shift has been observed for complexes (**1**)–(**4**). Furthermore, elemental analysis data have been useful to obtain empirical formulas of these complexes and to establish the purity of each complex.

Preliminary bioassays in antimicrobial activity showed moderate antibacterial activity with complexes (**1**) and (**2**) whereas ferrozine showed antifungal activity against *Candida albicans.* To the best of our knowledge, we are the first to report on the antifungal activity of ferrozine. These findings provide a potential lead for antimicrobial drug development.

## Figures and Tables

**Table 1:** FTIR data comparison chart of complexes (**1**)–(**4**) in comparison with those of free ligands.

	*ν*_C=N_ (cm^−1^)	*ν*_N=N_ (cm^−1^)
Ferrozine	1608	1503
Complex (**1**)	1596	1498
Complex (**2**)	1595	1498
Ferene	1589	1507
Complex (**3**)	1567	1499
Complex (**4**)	1570	1494

**Table 2:** Elemental analysis data of complexes.

Complex	Value	C (%)	H (%)	N (%)
(**1**)	Calculated	32.12	3.98	7.14
	Experimental	31.68	3.80	7.42

(**2**)	Calculated	39.66	3.25	9.03
	Experimental	39.29	3.76	9.23

(**3**)	Calculated	32.16	2.54	8.82
	Experimental	32.12	2.76	9.29

(**4**)	Calculated	33.78	1.89	9.55
	Experimental	33.76	2.42	9.58

**Table 3:** Mean inhibition zone diameter at 1 mg/disc of complexes (**1**) and (**2**) and at 20 *μ*g/disc of gentamicin.

	Mean inhibition zone diameter ± SEM (mm)	
	*S. aureus* ATCC® 25923	*E. coli* ATCC® 25922
Complex (**1**)	8.75 ± 0.75	7.50 ± 1.00
Complex (**2**)	7.00 ± 0.00	7.75 ± 0.25
Positive control (gentamicin)	26.00 ± 1.50	30.75 ± 0.75
Negative control	ND	ND

ND, not detected.

**Table 4:** Mean inhibition zone diameter for *Candida albicans* at 1 mg/disc of ferrozine and at 1 mg/disc of fluconazole.

	Mean inhibition zone diameter ± SEM (mm)
Ferrozine	13.00 ± 2.00
Fluconazole	29.75 ± 0.25

## Data Availability

The data used to support the findings of this study are included within the article and within the Supplentary Information file.

## Supplementary Materials

Supplementary Materials Table S1: comparison of UV-Vis data of ferrozine, ferene, and complexes (**1**)–(**4**) is presented in a tabulated form in Supplementary Materials.
