# Supplementary material for: Synthesis, Characterization, and Antimicrobial Activity of Novel Sulfonated Copper-Triazine Complexes
Source: Bioinorg Chem Appl. 2018 Aug 29;2018:2530851. doi: 10.1155/2018/2530851 (PMC6136538; doi:10.1155/2018/2530851)
Supplement: Supplementary Materials — Table S1: comparison of UV-Vis data of ferrozine, ferene, and complexes (1)–(4) is presented in a tabulated form in Supplementary Materials. [file 2530851.f1.docx]

**Synthesis, Characterization and Antimicrobial Activity of Novel Sulfonated Copper-triazine Complexes**

**Supplementary Information**

Table S1. Comparison of UV-Vis data of ferrozine, ferene, and complexes 1-4

| λ_ferrozine_ /nm | λ_Complex (1)_ /nm | λ_Complex (2)_  /nm | λ_Ferene_/nm | λ_Complex (3)_ /nm | λ_Complex (4)_ /nm |
| --- | --- | --- | --- | --- | --- |
| 211 | 205 | 213 | 209 | 202 | 208 |
| 236 | 242 | 240 | 243 | 239 | 246 |
| 285 | 298 | 301 | 306 | 338 | 338 |
| 310 | 327 | 334 | 339 | 371 | 371 |
